# On the transformation of “zincone”-like into porous ZnO thin films from sub-saturated plasma enhanced atomic layer deposition

**DOI:** 10.3762/bjnano.10.74

**Published:** 2019-03-21

**Authors:** Alberto Perrotta, Julian Pilz, Stefan Pachmajer, Antonella Milella, Anna Maria Coclite

**Affiliations:** 1 Institute of Solid State Physics, NAWI Graz, Graz University of Technology, Petersgasse 16, 8010 Graz, Austria; 2 Department of Chemistry, Università degli studi di Bari, Via E. Orabona 4, 70126, Bari, Italy

**Keywords:** calcination, PE-ALD, porosity, thin films, ZnO

## Abstract

The synthesis of nanoporous ZnO thin films is achieved through annealing of zinc-alkoxide (“zincone”-like) layers obtained by plasma-enhanced atomic layer deposition (PE-ALD). The zincone-like layers are deposited through sub-saturated PE-ALD adopting diethylzinc and O_2_ plasma with doses below self-limiting values. Nanoporous ZnO thin films were subsequently obtained by calcination of the zincone-like layers between 100–600 °C. Spectroscopic ellipsometry (SE) and X-ray diffraction (XRD) were adopted in situ during calcination to investigate the removal of carbon impurities, development of controlled porosity, and formation and growth of ZnO crystallites. The layers developed controlled nanoporosity in the range of 1–5%, with pore sizes between 0.27 and 2.00 nm as measured with ellipsometric porosimetry (EP), as a function of the plasma dose and post-annealing temperature. Moreover, the crystallinity and crystallite orientation could be tuned, ranging from a powder-like to a (100) preferential growth in the out-of-plane direction, as measured by synchrotron-radiation grazing incidence XRD. Calcination temperature ranges were identified in which pore formation and subsequent crystal growth occurred, giving insights in the manufacturing of nanoporous ZnO from Zn-based hybrid materials.

## Introduction

Atomic layer deposition (ALD) and molecular layer deposition (MLD) are sequential self-limiting vapor-phase deposition methods for the production of (ultra-)thin inorganic and organic films [1–2]. The layer-by-layer nature of these methods allows for the deposition of highly conformal thin films with sub-nanometer thickness control, making them the techniques of choice for complex high aspect ratio nanostructured substrates and devices.

By combining the metalorganic precursors adopted in ALD with the organic ligands used in MLD, hybrid organic–inorganic materials can be synthesized [2–4]. These materials possess properties in between their pure organic and inorganic counterparts, yet differing from the pristine materials [5–6]. In the literature, a wide array of hybrid films has been reported, adopting several metallic precursors (such as Al [7–10], Ti [11–12], Zr [11], Hf [13], Zn [14–17], V [18–19], Li [20], Fe [21–22], Mo [23] and Er [24]). Generally, the thin films comprise the metal element, oxygen, and an organic backbone, and are referred to as metal alkoxides or ‘metalcones’, e.g., alucones, zincones, and titanicones [2,4].

From the metal alkoxide produced with MLD, porous metal oxide thin films can be achieved through water etching or thermal treatments in the presence of oxygen. In the literature, (ultra-)thin porous alumina [7,25–29] and titania [12,30–33] are the most widely studied systems obtained from MLD metal alkoxides, and recently, vanadium-based materials were also investigated [18,34]. The porous thin films were applied as functional oxides in photocatalysis [30,33] and as electrodes for lithium-ion batteries [12,18], or for protective and passivating layers [28,32,35].

In this contribution, an alternative method was adopted for the formation of Zn-alkoxide layers. While ALD is known to deliver pure dense ZnO, applied in many technological fields [36–39], sub-saturated processes are generally not investigated. Starting from a saturated PE-ALD ZnO, optimized in a previous contribution [40], a sub-saturated ALD process was used to leave unreacted carbon contaminations and produce “zincone”-like layers with different organic fractions. The calcination of the hybrid layers was investigated in situ with spectroscopic ellipsometry (SE) and X-ray diffraction (XRD). Oxygen plasma was used as co-reactant together with diethylzinc (DEZ) in a room-temperature plasma-enhanced ALD process and, as a function of the plasma time exposure, the chemical composition of the Zn-alkoxide layers was controlled. In the literature, a C-content up to 7% was shown for oxides [41], sufficient to define these layers as Zn alkoxide or “zincone”-like. The effect of the combination of plasma exposure time and post-deposition calcination temperature on the formation of porosity was investigated with ellipsometric porosimetry (EP), already shown suitable for the determination of porosity in hybrid and polymer-derived oxides [12,18,29,42–43].

In the literature, few contributions investigate the transformation of zinc-based alkoxides into porous ZnO [15–16]. Liang et al. [15] reported on the transformation of zincones deposited on TiO_2_ nanoparticles into porous ZnO via thermal treatment in the presence of air. Low surface area and wide pore size distribution (in the micropore and mesopore size range) were achieved. The surface area and the content of mesopores were found to decrease with the post-deposition annealing temperature, which was attributed to clogging of the pores upon growth of ZnO crystals at relatively low temperatures. Thermogravimetric analyses were performed on the layers as a function of the post-deposition treatment up to 400 °C, showing significant loss of material starting from 200 °C. However, the crystallinity and crystal growth of the resulting porous ZnO structure were not investigated. In different systems, studying the evolution of Ti alkoxide upon pyrolysis in inert atmosphere, Abdulagatov et al. [11] showed an inhibition of the crystal formation in the resulting TiO_2_ layers, and the authors attributed this to the amorphous titania being constrained in the organic matrix. As a consequence, an investigation of the crystallinity of Zn-alkoxide layers would shed a light on the evolution of the ZnO crystals and, in turn, on the consequent possible clogging of the forming pores. Moreover, there are thorough investigations on the formation of porous oxides and metal-oxide/carbon composite films starting from MLD-derived metal alkoxides for Al-based [8,29] and Ti-based [11] materials. In these studies, process windows for the formation of highly porous materials are presented. Similar studies are missing for Zn-based materials.

## Experimental

A custom-built direct plasma ALD reactor was used to deposit the zincone-like thin films on one-side polished *c*-Si (100) substrates (Siegert Wafer). The reactor was in an asymmetrical plate configuration, in which the showerhead radio frequency (RF, at 13.56 MHz) electrode and ground electrode had a distance of 8 cm. Diethylzinc (DEZ, Sigma-Aldrich, CAS 557-20-0) was used as the metalorganic precursor. Pure oxygen (air liquide, 99.9995%) was used during the plasma step and Ar (air liquide, 99.9999%) was adopted in the purging step. The flow rates for O_2_ and Ar were set at 20 sccm during the plasma and the purging step, respectively. The O_2_ pressure in the reactor during plasma exposure was fixed at 85 mTorr. A multi gas controller (MKS 647C) and mass flow controllers (MKS MF1-C) were used to control the flow rates of the gases. An ALD-valve (Swagelok ALD3) was used to pulse DEZ into the reactor. Due to the high vapor pressure of DEZ, no further heating or bubbling system were adopted. All depositions were carried out at floating substrate temperature [40].

Spectroscopic ellipsometry (J.A. Woollam M-2000V) was used to deter0mine the thickness and optical properties of the films after deposition. The measurements were carried out at three different angles (65°, 70°, and 75°) in a wavelength range from 370 to 1000 nm. The analysis of the spectra was performed with the software CompleteEASE^®^. The thickness was determined by applying a three-layer model consisting of a silicon substrate, a native silicon-oxide layer with a fixed thickness of 1.5–2.0 nm, and a Cauchy layer, as follows

[1] $$n\left(\lambda \right)=A+\frac{B}{\lambda^{2}}+\frac{C}{\lambda^{4}}\;,$$

in which *n* is the wavelength-dependent refractive index, λ is the wavelength and *A*, *B*, and *C* are fit parameters. An Urbach tail was used to account for absorption at lower wavelength.

For the in situ temperature-dependent studies, the system was equipped with a THMS600 temperature stage (Linkam, UK), equipped with a sealing capping chamber. The temperature was varied from room temperature to 600 °C at 30 °C/min. The temperature was then kept constant at 600 °C for 100 min. Subsequently, the system was let cool down to temperatures of 30–40 °C, at the same rate. For these experiments, the acquisition angle was fixed at 70°.

For the determination of the porosity in the porous ZnO layers, the THMS600 temperature stage was equipped with a custom-made humidity pump, and the principles of ellipsometric porosimetry (EP) were used. The relative humidity was monitored via a sensor in the measurement chamber (Sparkfun SHT-15) and tuned in the range of 0–95% [44]. In this way, pores with a diameter ≥0.33 nm were probed. Dry atmosphere (i.e., 0% relative humidity) was reached using a flow of N_2_, ensuring the removal of water from the measurement chamber. In order to tune the relative humidity, the flow of humid air is mixed with N_2_ and adjusted in equilibrium steps. The temperature of the stage was kept at room temperature, assuring that the measured relative humidity in the chamber was the same as on top of the samples.

During EP with water, the variation of both refractive index (*n*) and water multilayer thickness/swollen thin films is reported as a function of the relative humidity, resulting in classical adsorption/desorption isotherms, generally categorized according to the IUPAC classification [45–48]. A type-I isotherm is associated with porous materials with a narrow distribution of pore size with a diameter below 2 nm (nanoporous or microporous materials), and a type-II isotherm is associated with nonporous materials. Mesoporous materials are instead characterized by a type-IV isotherm, in which a hysteresis arises in the desorption step, due to the condensation of water in the pores. The adsorptive uptake was expressed as adsorptive volume obtained from the Lorentz–Lorenz relationship and calculated as:

[2] $$V_{\text{ads}}/V_{\text{film}}=V_{\text{mol}}/\left[\alpha_{\text{ads}}\cdot \left(d_{\text{t}}+d_{0}\right)\right]\cdot \left(\left(B_{\text{s}}d_{0}+B_{\text{t}}d_{\text{t}}\right)-B_{0}d_{0}\right)\;,$$

where *V*_ads_ is the volume of the liquid adsorptive in pores, *B*_0_ and *B*_s_ are the volume polarizability of the film before and during adsorption, *B*_t_ is the volume polarizability of the adsorbate multilayer that develops on top of the layer, *d*_0_ and *d*_t_ are the thickness of the layer and the adsorbate multilayer, respectively, *V*_mol_ is the molecular volume of the adsorptive, and α_ads_ is the polarizability of the adsorptive molecule. If swelling occurs, *d*_t_ is ignored in the equation and only the layer thickness is taken into account, as reported in [49]. Therefore, the adsorptive volume contains both information about pore filling and the formation of multilayers or swelling.

The porosity (*P*) in the layers is calculated from the isotherm when all the pores accessible to the probe molecules have been filled. Using the Lorentz–Lorenz equation, *P* can be expressed as:

[3] $$P=\left[\frac{\left(n_{\text{fill}}{}^{2}-1\right)}{\left(n_{\text{fill}}{}^{2}+2\right)}-\frac{\left(n_{0}{}^{2}-1\right)}{\left(n_{0}{}^{2}+2\right)}\right]/\frac{\left(n_{\text{water vapor}}{}^{2}-1\right)}{\left(n_{\text{water vapor}}{}^{2}+2\right)}\;,$$

in which *n*_0_ and *n*_fill_ are the refractive index values of the layer when the pores are empty and filled, respectively, and *n*_water vapor_ is the water refractive index. With this approach, the porosity values obtained are independent of the refractive index of the matrix. More details on the technique can be found in [50–51].

X-ray diffraction (XRD) was performed to analyze the crystalline properties of the films in specular direction, that is, with the crystallographic planes parallel to the substrate. The diffractometer (Panalytical Empyrean), working in a θ/2θ-configuration, was equipped with a copper tube (λ = 1.5418 Å). The beam was further parallelized with a layered X-ray mirror and a PIXcel^3D^-detector that was operated in 1D-mode. A 1/8° divergence, a 10 mm mask, and a P7.5 anti-scatter slit were used in the setup. In situ temperature-dependent XRD studies were performed with a DHS900 heating stage attachment (Anton-Paar, Austria), using a heating rate of 30 °C/min up to 600 °C. During the ramp, samples were kept at 200, 300, 400 and 600 °C for 30 min each. The integration time was set to 3 min per measurement [52]. All data were recorded using the same setup and are represented in the scattering vector (*q_z_*) notation, where *q_z_* = 4π·sin(θ)/λ.

Grazing incidence X-ray diffraction (GIXD) was performed to investigate the in-plane orientation of the crystallites, that is, with the crystallographic planes perpendicular to the substrate. The measurements were conducted at the Elettra XRD1-beamline in Trieste, Italy [53]. The wavelength for the primary beam was set at 1.4 Å, under an incident angle α of 1.2–2.0°. Diffracted intensities were collected on a Pilatus 2M detector and all data have been recalculated to (wavelength-independent) reciprocal space maps using the in-house developed software package GidVis [54]. Intensities are plotted in a pseudo-color representation as a function of the specular (*q_z_*) and the in-plane component (*q_xy_*) of the scattering vector. For the sake of clarity and comparability, all intensity data were plotted in square root representation and are reported using the same color scales [55].

For chemical analysis of the obtained thin films, Fourier-transform infrared (FTIR) and X-ray photoelectron spectroscopy (XPS) were performed. For the FTIR analysis, a Bruker IFS 66v/S was adopted. For each spectrum, 1000 scans were recorded in transmission mode with a resolution of 4 cm^−1^ between 400 cm^−1^ and 4000 cm^−1^. All spectra were baseline corrected and normalized to the film thickness.

Surface chemical composition was investigated by X-ray photoelectron spectroscopy (XPS). Analyses were performed with a Scanning XPS Microprobe (PHI 5000 Versa Probe II, Physical Electronics), equipped with a monochromatic Al Kα X-ray source (1486.6 eV) operated at 15 kV with a spot size of 100 µm and a power of 24.8 W. Survey (0–1200 eV) and high resolution (HR) spectra were recorded in FAT (Fixed Analyzer Transmission) mode at a pass energy of 117.40 and 29.35 eV, respectively. Under the set conditions, the energy resolution of the analyzer (FWHM, full width at half-maximum height), measured on the silver Ag 3d_5/2_ photoemission line, was 0.7 eV for a pass energy of 29.35 eV. All spectra were acquired at a take-off angle of 45° with respect to the sample surface. Surface charging was compensated using a dual beam charge neutralization, with a flux of low-energy electrons (ca. 1 eV) combined with positive Ar ions of very low energy (10 eV). Samples were sputter cleaned for 1 min with an Ar ion beam of 1 kV, 1 µA (raster size: 2 × 2 cm^2^). The acquired spectra were processed with CasaXPS software. The lattice O–Zn component of the O 1s spectrum was used as internal standard for charging correction and it was set to 529.8 eV [56].

## Results and Discussion

### Optochemical characterization of the zincone-like layers

**Figure 1:**
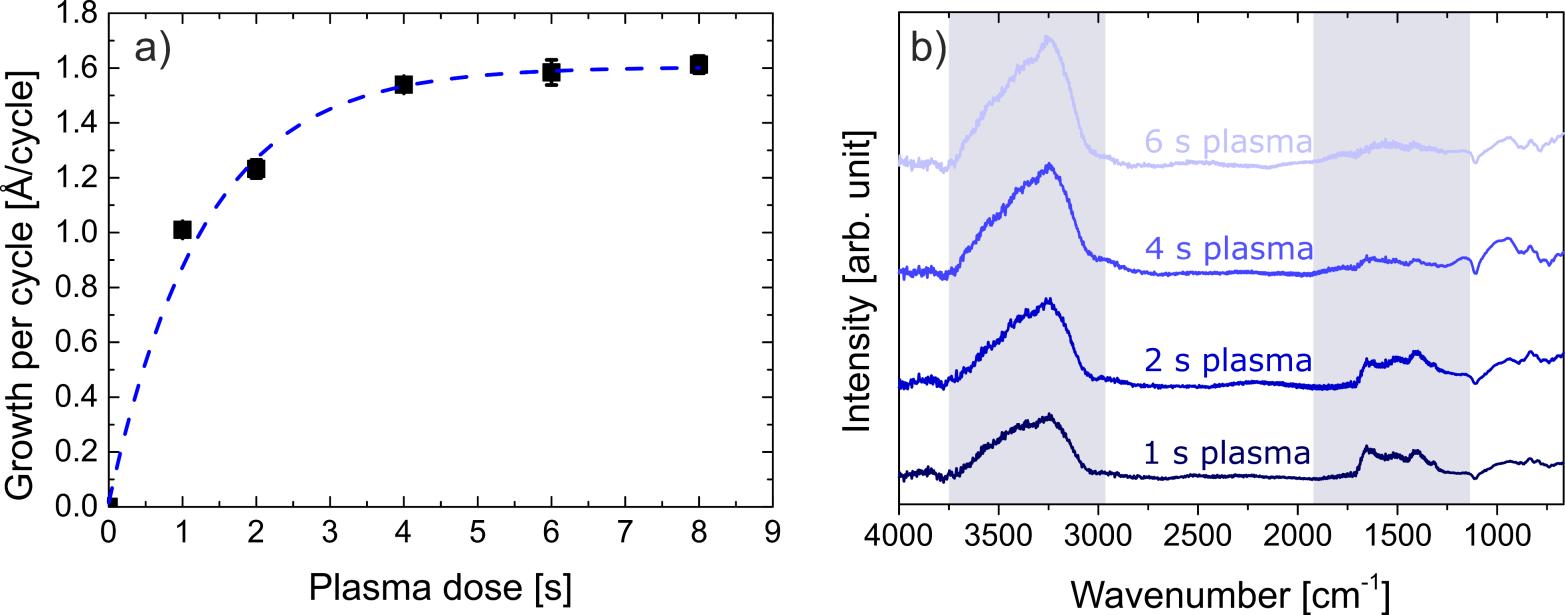
a) Growth per cycle (GPC) as a function of the plasma dose time. The saturation of the PE-ALD ZnO recipe was reached at 6 s of plasma exposure. Error bars are plotted for all data points but are within marker size for most points. b) FTIR spectra of the zincone-like layers deposited at different plasma pulse time. The spectra are offset for clarity. The main absorption regions attributed to the ν_OH_ (3000–3600 cm^−1^) and to the stretching of carbonate/bidentate/carboxyl groups (ν_OCO_) (1300–1660 cm^−1^) are highlighted in the image.

Zincone-like layers were deposited by varying the plasma pulse time between 1 and 6 s, with the latter being the condition at which saturation occurred. DEZ pulse time and purging times (for DEZ and O_2_ plasma) were kept constant and under saturation condition, that is, 150 ms, 12 s, and 15 s, respectively (see Supporting Information File 1). The growth per cycle (GPC) as a function of the plasma dose time is reported in Figure 1a. The GPC was found to saturate at 1.6 Å/cycle, which is in line with the literature values for processes carried out at room temperature and the ones previously reported [40,57]. Plasma dose times below 6 s led to lower GPC, as expected, down to values of 1.0 Å/cycle for the plasma doses explored. The lower GPC resulted from the partial removal of the organic ligands of the metalorganic precursor (DEZ) and the formation of a limited number of active groups able to react with the next metalorganic precursor exposure.

In order to gather information about the chemical composition and bonding of the Zn-alkoxide layers, FTIR and XPS measurements were performed. The oxidative environment of the oxygen plasma during the deposition causes the formation of CO_2_ and water eventually leading to the removal of all the carbon functionalities and formation of pure ZnO. Before saturation, though, the active oxygen species are not sufficient in number to cause the total reaction of the ethyl groups into CO_2_, leaving in the layer (partially) oxidized organic groups and chain terminating Zn–OH groups. In Figure 1b, the FTIR spectra are reported as a function of the plasma dose. Two characteristics absorptions were identified. The stretching (ν) of hydroxyl (–OH) moieties was present in the spectra for all layers at wavenumbers in the range 3000–3600 cm^−1^. The ν_OH_ intensity was found to increase as a function of the plasma dose. This was justified with the formation of more Zn–OH groups due to the removal of the organic ligands. Furthermore, room-temperature PE-ALD processes have often shown in the literature the inclusion of chain-terminating –OH groups in the inorganic matrix, due to the low energy provided during the growth [47,57]. The presence of organic functionalities is confirmed by the absorption in the region of 1300–1600 cm^−1^. The absorption in this region can be assigned to (a)symmetric (bidentate) OCO stretching vibrations in bicarbonates, carbonates, and formates (ν_OCO_) due to the partial oxidation of the organic ligands. These absorptions have been previously reported for both low-temperature and high-temperature PE-ALD processes [51,57] and as intermediates in in situ FTIR studies [58]. The absence of IR absorption in the region of 3000–2800 cm^−1^ points out the complete oxidation or partial removal of the ethyl groups present in the pristine DEZ precursor molecule. As a function of the plasma dose time, the ν_OCO_ absorption was found to decrease and to disappear at 6 s plasma exposure time. This infers a different organic fraction in the layers and the formation of ZnO under saturation conditions. Moreover, the increase in the –OH absorption has to be attributed to increased formation of Zn–OH groups as a function of the plasma time.

**Figure 2:**
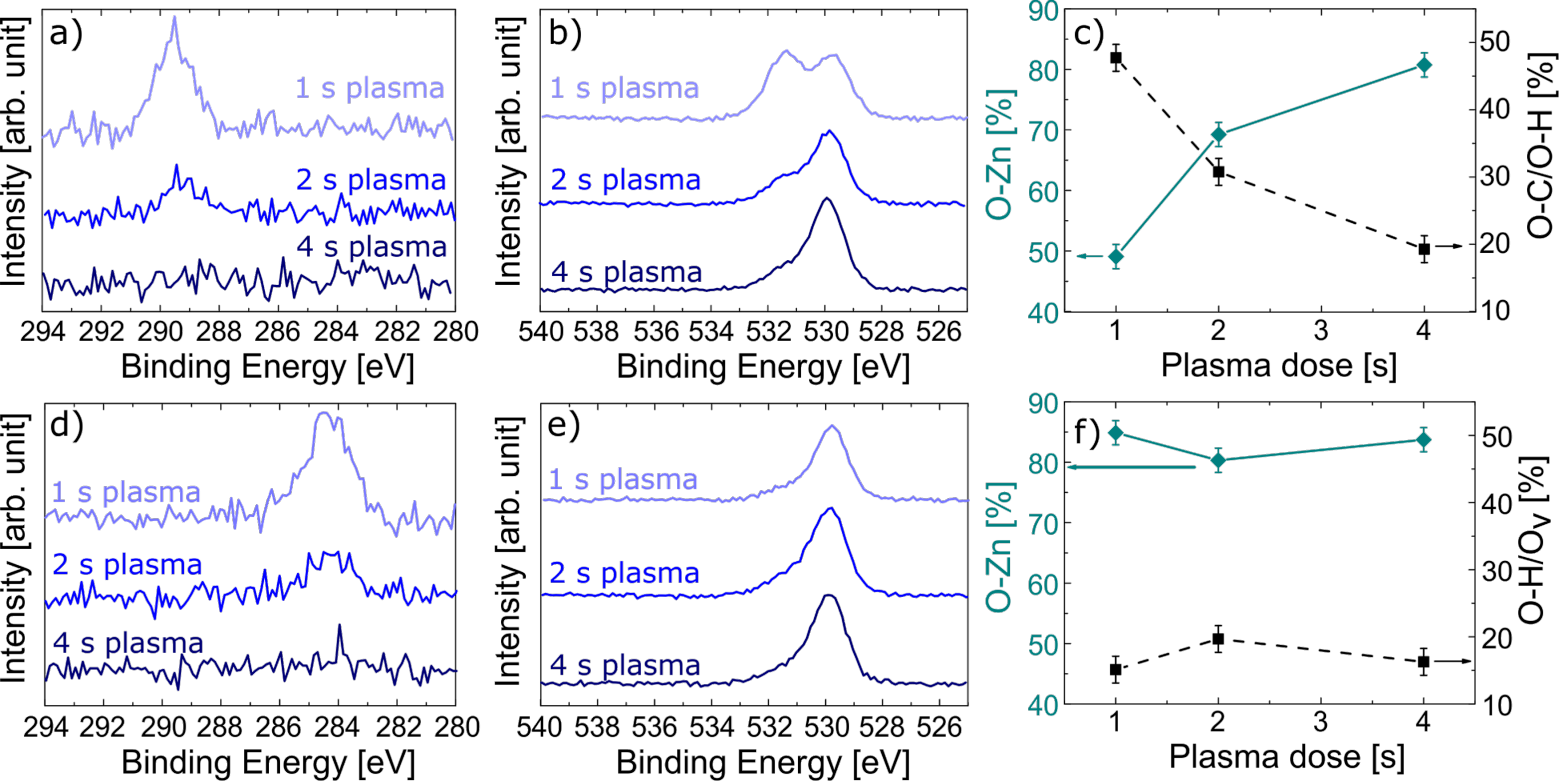
High-resolution XPS spectra of the pristine (a–c) and annealed (d,e) zincone-like layers acquired after 60 s Ar^+^ sputtering. Pristine: a) C 1s HR spectrum and b) O 1s HR spectrum as a function of the plasma pulse time. c) Relative amount of the O–Zn and O–C/O–H as a function of the plasma pulse time as resulted from the O 1s peak fitting. Annealed: d) C 1s spectrum and e) O 1s spectrum as a function of the plasma pulse time. f) Relative amount of the lattice O–Zn and Zn hydroxides/defective oxygen as a function of the plasma pulse time, as resulted from the O 1s peak fitting.

In Figure 2, the high-resolution XPS spectra of C 1s and O 1s are reported as a function of the plasma exposure time. In Table 1, the atomic content (atom %) of carbon, oxygen, and zinc are also reported.

**Table 1:** Carbon, oxygen, and zinc atomic percentage (atom %) as measured by XPS for the zincone-like layers as a function of the plasma dose time. O/Zn ratios are also reported. Values before and after the annealing procedure are reported for all the elements. In brackets, the O/Zn calculated from the value of pure bulk ZnO obtained from reference [58].

Plasma pulse [s]	C [atom %] (±1%)		O [atom %] (±1%)		Zn [atom %] (±1%)		O/Zn (±0.1)		*n* (at 633 nm) (±0.003)
	before	after	before	after	before	after	before	after	before

1	7	13	45	35	48	52	0.9 (1.3)	0.7 (1.0)	1.664
2	4	4	41	42	55	54	0.8 (1.1)	0.8 (1.1)	1.713
4	2	<1	41	41	57	58	0.7 (1.0)	0.7 (1.0)	1.835

As clearly shown in Figure 2a, all carbon atoms present in the zincone-like layers are fully oxidized. Peaks assigned to the C–C bonds (284.8 eV) were missing in all layers. Instead, a peak at 289.5 eV was present, indicative of C(O)OH/C(O)OR groups [59], thus confirming the conclusions drawn from the FTIR spectra. The C content decreased as a function of the plasma exposure time, ranging from 7% at 1 s plasma dose to 2% at 4 s plasma dose (Table 1), in line with the literature values obtained for PE-ALD unsaturated processes at room temperature [41]. In Figure 2b, the O 1s HR spectra are reported as a function of the plasma exposure time (see also Supporting Information File 1). For short plasma exposure times, the spectrum was fitted with three distinct peaks: one at 532.8 eV, attributed to organic oxygen, another one at 531.3 eV, assigned to Zn hydroxides and/or defective oxygen (vacancies). The latter peak is also attributed to the formation of metal carbonates, in line with the FTIR results and the literature values [60]. Finally, the peak at 529.8 eV was attributed to lattice O–Zn bonds [56].

Increasing the plasma time led to the decrease and eventual disappearance of the pure organic oxygen peak and the decrease of the peak associated to Zn hydroxides and carbonates. The relative content of the components is reported in Figure 2c. Increasing the plasma exposure time, a higher content of Zn–O lattice bonds is formed, eventually reaching values close to high-quality ZnO deposited at room temperature [40]. The O/Zn ratio is also reported in Table 1. All values are below unity, due to the preferential sputtering occurring when atomic masses of the measured elements are very different, which is the case of Zn and O. In the literature [61], the O/Zn ratios are generally normalized to the values obtained from bulk crystalline ZnO, in the order of 0.71. Using this value as a reference, in Table 1, corrected O/Zn ratio are reported in brackets and will be referred to in the discussion. At low plasma exposure time, O/Zn ratios of 1.3 were found. The higher amount of oxygen was attributed to the presence of oxidized carbon species, increasing the oxygen atomic content. As the plasma dose time was increased, the O/Zn ratio converged to values close to unity.

The layers were also characterized by means of SE. The refractive index *n* as a function of the plasma exposure time (Table 1) confirmed the trends inferred by XPS and FTIR. After short exposure to plasma, lower refractive index values were obtained due to the presence of higher amounts of carbon and, in turn, lower density of the material. Increasing the plasma exposure time led to an increase of the refractive index, close to the one obtained under saturation conditions at room temperature (1.858 ± 0.003 at 633 nm).

In summary, the optochemical characterization of the layers confirmed the deposition of layers with a different organic fraction (Zn-alkoxide or zincone-like) and different chemical environments. The calcination of thse layers in air would lead to the removal of the organic functionalities and, in turn, to the possible formation of pores as a function of the C-content.

### Calcination in air

#### Spectroscopic ellipsometry

**Figure 3:**
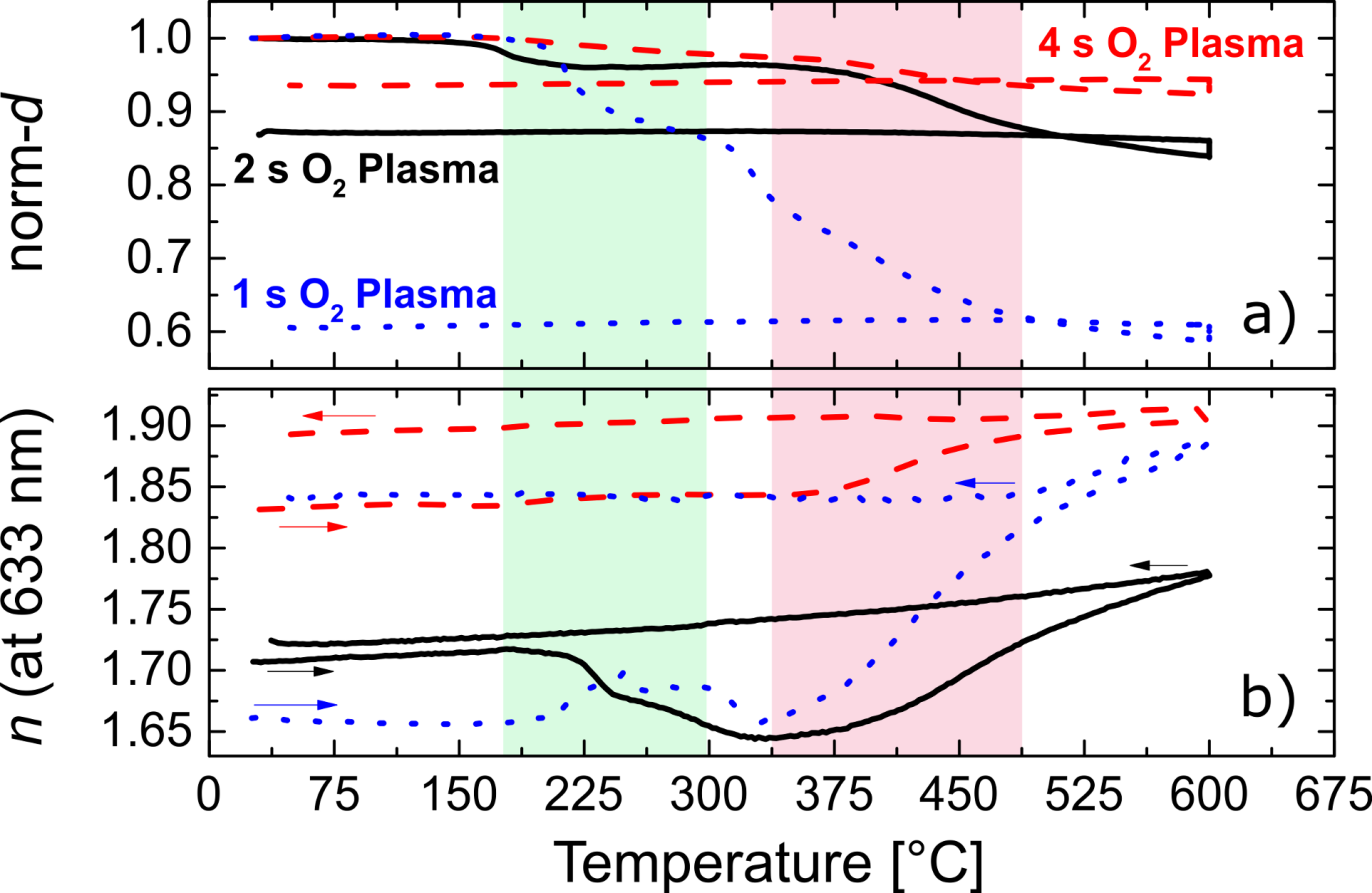
a) Normalized thickness (norm-*d*) and b) refractive index *n* (at 633 nm) of the zincone-like layers as a function of the annealing temperature. The two critical temperature ranges for initial removal of organic moieties and growth of ZnO crystals are highlighted. The two parameters were modeled from the SE data acquired in situ during the annealing of the thin films. The thickness is reported as normalized to the initial value (30 ± 3 nm) of every layer for clarity.

In order to monitor in situ the evolution of the zincone-like layers when calcined in air, SE and XRD measurements were performed during the heating and cooling of the samples. In Figure 3, the thickness and refractive index of the layers obtained by modelling of SE measurements are reported as a function of the annealing temperature.

In terms of thickness variation, two distinct regions can be identified for all the plasma conditions adopted during deposition. By increasing the temperature up to 170 °C, the layers were stable and no variation in thickness was witnessed. In the temperature range of 170–270 °C, an overall decrease in thickness was observed, due to the removal of C impurities. Consequently, the relative thickness loss was found to depend on the plasma exposure time. Layers deposited with long plasma exposures (4 s) showed a relative initial thickness loss of 2.6 ± 0.2%, while layers deposited with 1 s plasma exposure showed a thickness decrease of 12.8 ± 0.2%. In the same temperature range, the refractive index showed different behaviors as a function of the plasma exposure time. The refractive index can give important information on the possible formation of porosity in the layers. At 4 s of exposure, a slight increase of 0.01 in refractive index was measured. The carbon concentration after deposition (2%, Table 1) is not enough to induce major changes in the refractive index when partially removed. Furthermore, the thickness loss (<1 nm) is of the order of the adventitious carbon present on the surface of the layer due to exposure to atmosphere. For the layers deposited with 2 s of plasma exposure, a strong drop in *n* was measured between 225 and 300 °C, with values down to 1.644 ± 0.003. In this temperature region, the layer thickness stayed constant, indicating the absence of collapsing of the non-volatile backbone, and, in turn, formation of voids in the material accounting for the lower refractive index. When the plasma exposure is instead very short (1 s), an increase of the refractive index was measured in the first critical temperature region, up to 1.704 ± 0.003. The densification of the layer, together with the 12.8% of thickness loss, suggests a collapse of the backbone of the zincone-like layer.

A further increase in temperature identifies a second critical region, between 350 and 470 °C. In this range, the thickness decreased further for all layers under investigation. As a function of the plasma exposure time, the total thickness loss ranged from 7.2% (4 s plasma exposure) to 41% (1 s plasma exposure). Similar results were reported by Liang et al. [15] for MLD zincone layers. Material loss as measured with thermogravimetric analysis was reported in similar temperature ranges, confirming the similarities between the MLD layers and the PE-ALD zincone-like films reported here. The refractive index was instead found to increase with the annealing temperature. Two distinct regions can be identified for both thickness and refractive index variation. At first, a steep decrease in thickness was witnessed in a temperature range depending on the C-content of the layers (Figure 3a). For the layers with the highest C-content, the sharp thickness decrease occurred between 310 and 490 °C. In this temperature range, the refractive index increased for all the layers (Figure 3b). At higher temperatures, a less steep slope was found both for the decrease of thickness and increase in refractive index. Two mechanisms could account for such phenomena. Residual carbon present in the layers is oxidized and desorbed, causing a further collapse of the non-volatile ZnO backbone. In an in situ FTIR investigation of the pyrolysis of alucone deposited by MLD, Dumont and George [8] reported on the disappearance of the organic absorption bands as a function of the annealing temperature. The C-containing moieties were found mostly to disappear in the range of 400–550 °C. However, in this investigation FTIR measurements between the deposition (150 °C) and the first annealing temperature (400 °C) were not reported. The disappearance of C-moieties seems linear as a function of temperature, suggesting a continuous oxidation/removal process. In the present case, the organic fraction is already partly oxidized, decreasing the amount of thermal energy necessary to complete the oxidation and the removal of carbon from the layers. Nevertheless, considering the substantial thickness decrease (41%) of the ZnO layers (1 s of exposure), this mechanism is reasonable. The second simultaneous mechanism is attributed to the crystal formation/recrystallization of ZnO. In the literature, post-deposition annealing studies on ALD ZnO layers [62] reported an increase of refractive index between 400 and 700 °C. The increase was attributed to the growth and coalescence of the ZnO crystallites. In the present contribution, similar conclusions were drawn based on the XRD measurements, discussed later in the paper, and accounting for the increase in refractive index as measured by SE. For low C-content layers (4 s plasma exposure) a collapse of the backbone is instead unlikely, due to the very low initial carbon fraction. In this case, the increase of the refractive index measured in the range of 375–475 °C is solely attributed to the growth of ZnO crystals.

#### X-ray photoelectron spectroscopy

In Figure 2d–f, the XPS measurements performed on the zincone-like layers after calcination are reported. In Table 1, the atom % fractions after calcination are specified. In Figure 2d and reported in Table 1, the C 1s HR spectra show the presence of non-oxidized carbon, at 284.2 eV, with C fractions higher than in the pristine layer, likely stemming from impurities and adventitious carbon diffusing in the layers during the calcination. The formation of graphitic nanocrystalline domains, previously reported for pyrolyzed titanicone layers [11], can be excluded, considering that annealing was performed in an oxidative environment (air). The absence of oxidized carbon confirms the successful calcination and the removal of the residual organic ligands in the layers. In Figure 2e, the O 1s HR spectra confirm the removal of the carbon functionalities and the calcined layers showed an O/Zn ratio of 1.0 ± 0.1 (Table 1). The oxygen peaks were decomposed into two contributions, assigned to the oxygen–zinc bonds in the oxide lattice (O–Zn, 529.8 eV), and zinc hydroxides and/or oxygen vacancies (Zn–OH/O_v_, 531.4 eV) [60,63]. As reported in Figure 2f, the Zn–OH/O_v_ component was found to decrease after calcination, with values resembling those of pure ZnO deposited at room temperature under saturation conditions [40]. The annealing step in the presence of oxygen leads to the formation of ZnO with fewer defects. In contrast, the layers deposited at long plasma exposure times, having a very low C-content, did not develop sufficient amounts of new ZnO to affect the Zn-OH/O_v_ component.

#### X-ray diffraction

**Figure 4:**
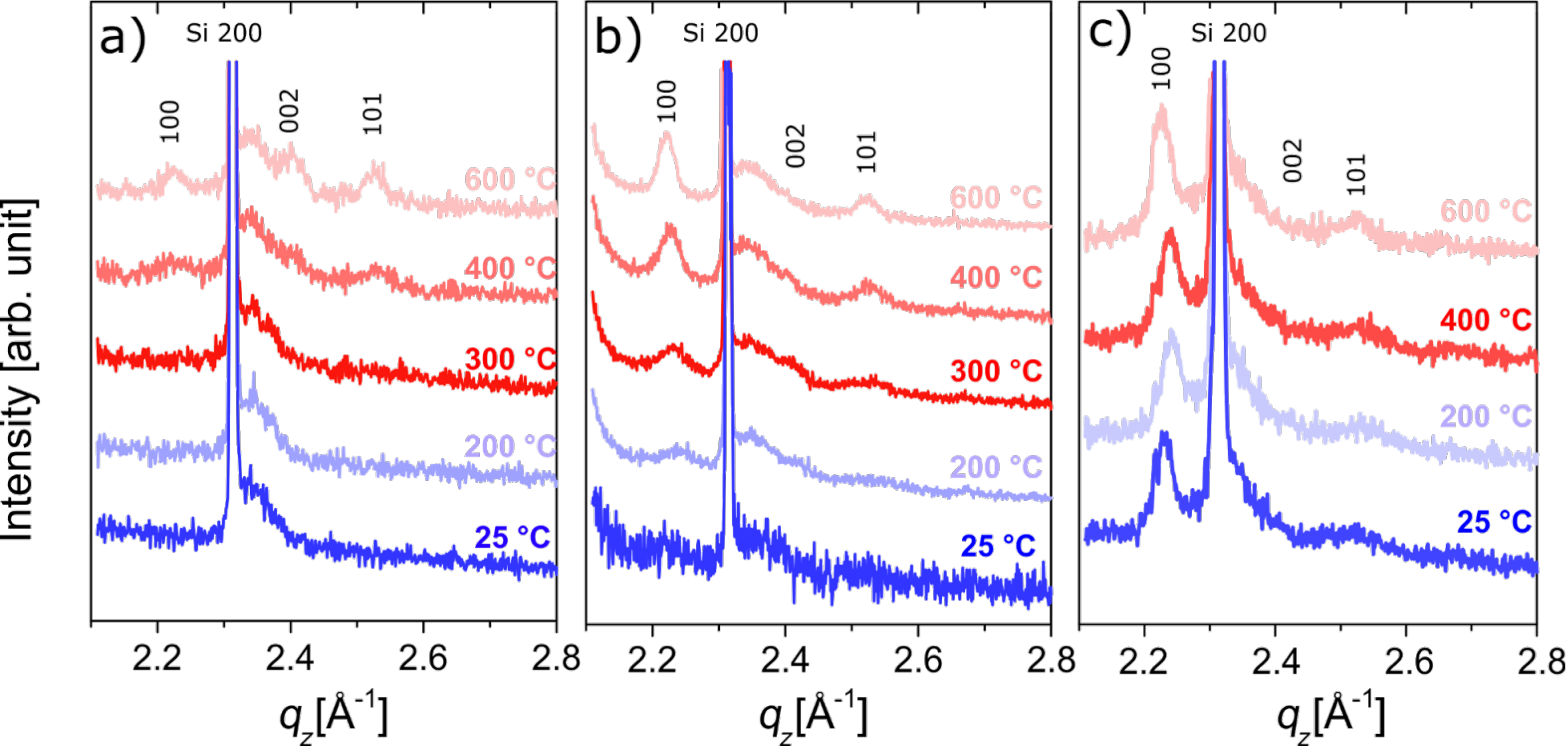
In situ XRD measurements performed during calcination between 25 and 600 °C for the zincone-like layers deposited with a) 1 s plasma exposure time, b) 2 s plasma exposure time, and c) 4 s plasma exposure time. Bragg peaks originating from ZnO and Si are indicated by their respective Miller indices.

As mentioned before, the preferential orientation of ZnO is paramount when a specific application is targeted, since its crystal growth and texture affect the optical and (piezo)electrical properties of the material [64–66]. The crystallinity of the pristine zincone-like layers and its evolution during and after calcination were monitored using in situ X-ray diffraction (XRD) and grazing incidence X-ray diffraction (GIXD). In Figure 4, the in situ XRD measurements performed during the calcination procedure for the zincone-like layers under investigation are presented.

In a reference ZnO powder (26170-ICSD [67]), peaks are generally observed at *q_z_* positions of 2.23 Å^−1^, 2.41 Å^−1^, and 2.54 Å^−1^, which match with the [100], [002], and [101] directions, respectively. The positions of the Bragg peaks are indicated in Figure 4. In addition, measurements are sorted from Figure 4a to Figure 4c as a function of plasma exposure time during sample preparation, i.e., from 1 s (Figure 4a) over 2 s (Figure 4b) to 4 s (Figure 4c). Because of the Si substrate, the Si 200 peak is visible in all diffractograms.

Starting with the spectra of the pristine Zn-alkoxide layers acquired at 25 °C before calcination (dark blue lines in Figure 4a–c), no ZnO peaks are observed for the sample deposited with 1 s plasma exposure time (dark blue line Figure 4a). The sample does not present any crystallinity as a consequence of the incomplete removal of the organic ligands, and the Bragg peaks present at 2.32 Å^−1^ are identified solely as Si(200). Already after a plasma exposure time of 2 s, broad peaks get visible at the (100) and (101) peak positions (dark blue line Figure 4b), inferring the formation of the first ZnO crystallites, and especially the (100) peak clearly evolves with an increased plasma exposure time of 4 s (dark blue line Figure 4c), resembling more the pure ZnO previously reported in [40]. From this, a control from amorphous zinc alkoxide (1 s plasma exposure) to crystalline ZnO (4 s plasma exposure) with a preferred (100) orientation as a function of plasma time can be concluded. During the calcination of samples deposited at 1 s plasma exposure time, ZnO peaks become visible starting from an annealing temperature of 400 °C at *q_z_* values of 2.21 Å^−1^, 2.40 Å^−1^, and 2.52 Å^−1^, respectively and further increase in intensity at 600 °C (Figure 4a, light blue to light red lines). This indicates the formation and growth of randomly oriented crystallites as the peak positions can be attributed to the [100], [002] and [101] directions of ZnO. Similar patterns were also measured for the zincone-like layers deposited at higher plasma times (c.f. Figure 4b and Figure 4c, light blue to light red lines). In this case, the Bragg peaks were found to increase in intensity also at lower temperatures, starting from 300 °C, most likely due to the coalescence of ZnO crystallites already formed during the deposition process at room temperature. However, the intensity was found to mostly increase when the calcination temperature reached 400 °C. As a function of the plasma exposure time during deposition, different preferential orientations were measured during the calcination procedure. At low plasma time (1 s), the relative intensity of the (100), (002), and (101) peaks increases steadily with annealing temperature, suggesting a random orientation in the film. At long plasma exposure times (4 s), the preferred orientation is evident at room temperature and the (100) peak increases in intensity with the calcination temperature. At 2 s plasma exposure time, while the (100) peak still grows in intensity faster than the other Bragg peaks, the (002) and (101) peaks become sharper and more intense with the calcination temperature, suggesting a different preferential orientation of the ZnO grown under these conditions.

**Figure 5:**
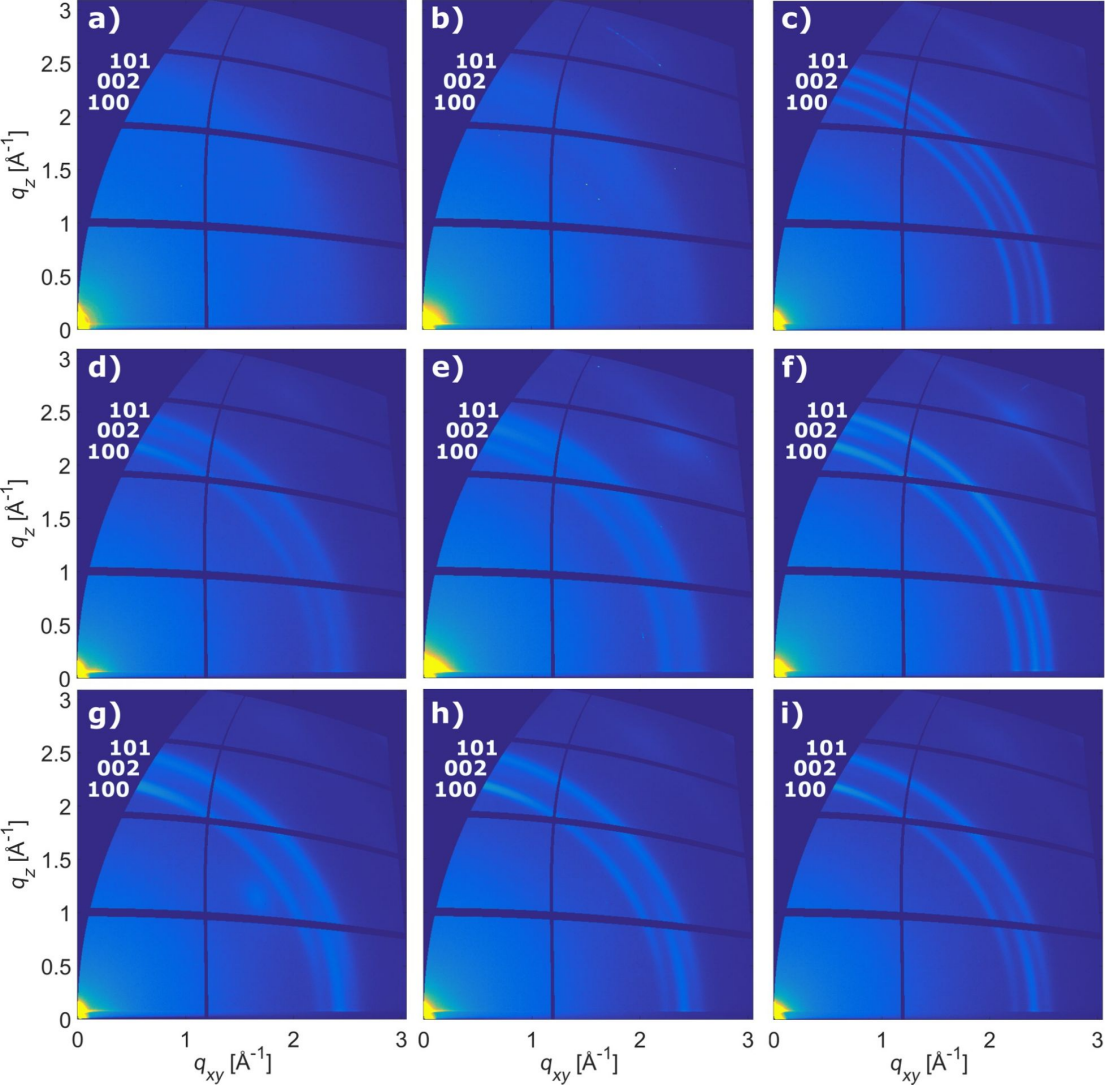
GIXD maps for the zincone-like layers deposited at different plasma times before and after annealing at different temperatures: 1 s plasma exposure time a) 25 °C, b) after 200 °C calcination, c) after 400 °C calcination; 2 s plasma exposure time d) 25 °C, e) after 200 °C calcination, f) after 400 °C calcination; 4 s plasma exposure time g) 25 °C, h) after 200 °C calcination, i) after 400 °C calcination.

In order to gather information also on the in-plane orientation of the ZnO crystallites, ex situ GIXD maps were acquired at synchrotron Elettra, Trieste, and are presented in Figure 5. Data are represented as reciprocal space maps, where the out-of-plane scattering vector *q_z_* is plotted over the in-plane scattering vector *q_xy_*. The colormap of the intensity ranges from dark blue (low intensity) to bright yellow (high intensity). Close to the coordinate origin, high intensity due to the primary beam is visible in all measurements. Please note that due to geometrical restrictions the pure specular direction *q_xy_* = 0 is not available (dark blue area).

To start with the pristine zincone-like layers, the measurements for the samples prepared with 1 s plasma exposure time, 2 s plasma exposure time and 4 s plasma exposure time are reported in Figure 5a, Figure 5d and Figure 5g, respectively. For the sample deposited with 1 s plasma exposure time (Figure 5a), no diffraction signal was measured, indicating an amorphous material. In the sample deposited with 2 s plasma exposure time (Figure 5d), diffraction signals appear along Debye–Scherrer rings which can be correlated to the (100), (002) and (101) Bragg peaks of ZnO. For the (002) ring, intensity is only present close to the out-of-plane direction and close to the in-plane direction, respectively. Having the wurtzite structure of ZnO in mind, this indicates standing and lying crystallites with (002) orientation within the same sample, although the distribution of the intensity along the ring indicates a high mosaicity. This mosaicity together with the amount of planes equivalent to the (100) and (101) planes explains the presence of intensity along the whole rings for these peaks. The sample prepared with a plasma exposure time of 4 s (Figure 5g) behaves in a similar fashion, but with the (002) Bragg peak now present only in-plane. Together with this, also the (100) and (101) rings lose intensity close to *q_z_* = 0. Both confirms a preferential out-of-plane texture of the crystallites in the [100] direction for longer plasma exposure times during deposition.

For the calcined samples, in the literature major changes are reported in the optical and morphological properties of pure ZnO thin films deposited with different methods in the annealing temperature range of 350–450 °C [62,68–73]. For the pyrolysis of titanicone, a shift towards higher crystallization temperatures for TiO_2_ was reported by Abdulagatov et al. [11] due to hindrance of crystallization by organic ligands constraining amorphous TiO_2_ in a carbon matrix. For the zincone-like layers reported here, due to the low organic fraction, the oxidizing atmosphere, and the lower crystallization temperature of ZnO compared to TiO_2_, the properties of pure ZnO are still maintained. While for the samples annealed at 200 °C (Figure 5b,e,h) almost no improvement is visible, the appearance of defined and sharper Debye–Scherrer rings due to crystal growth was measured starting from 400 °C for all investigated layers (Figure 5c,f,i). GIXD maps of the layers annealed at 600 °C are presented in Supporting Information File 1. One should note that the orientation distribution of crystallites, tuned by the plasma exposure time during sample preparation, is maintained while annealing and the new ZnO crystals grow with the same orientation as the previously formed crystals. Samples prepared with 1 s plasma exposure time crystallized from the amorphous state into a powder-like diffraction pattern (Figure 5b). Samples deposited with 2 s plasma exposure time kept both standing and lying crystallites, i.e., the ZnO crystallites are with their (001) planes either parallel or normal oriented to the substrate surface. Samples prepared with 4 s plasma exposure time finally show a clear preferred orientation with their (001) planes normal to the substrate surface.

Overall, the GIXD measurements of the pristine zincone-like layers confirm the results obtained with in situ XRD and especially verify the tunability of Zn-alkoxide layers towards out-of-plane oriented crystallites with the (100) plane parallel to the substrate. Moreover, in the XRD diffractograms as well as in the reciprocal space maps the peaks of all the zincone-like layers became sharper and more intense at 400 °C, confirming the crystal growth and accounting for the increase in refractive index as measured with SE. It can therefore be concluded that the combination of plasma exposure time and annealing allows for the tuning of the final texture of the resulting ZnO.

**Figure 6:**
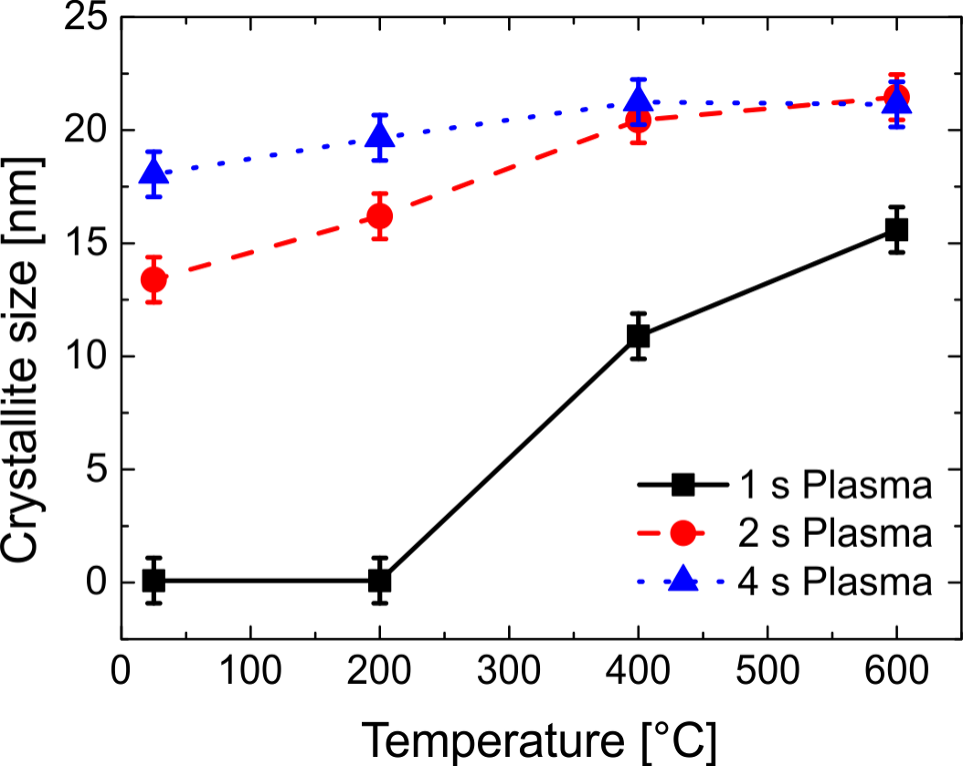
Estimated crystallite size *D* in the [100] direction as a function of the annealing temperature for the zincone-like layers deposited at different plasma times.

Furthermore, the temperature range of 350–450 °C would be crucial in the formation and the clogging of porosity. Liang et al. [15] reported on the formation of mesoporous and microporous layers on nanoparticles coated with MLD zincone layers. In their contribution, the pore size distribution and surface area were reported as functions of the calcination temperature, showing a decrease in the overall porosity starting from 350 °C. They attributed this effect to the formation of ZnO crystals, clogging the pores. For the zincone-like layers presented here, in order to quantify the crystal growth, an estimation of the average crystallite size *D* was performed. *D* can be obtained from a Bragg peak in the XRD spectrum using the Scherrer equation [74],

[4] $$D\approx \frac{\lambda }{\beta_{2\theta }\cos\theta }\;,$$

where λ is the wavelength of the X-rays, β_2θ_ is the full-width at half maximum of the peak, and θ is the peak position. The crystallite size for the (100) peak at different annealing temperatures for the zincone-like layers is presented in Figure 6.

The overall crystallite dimension reached a limit coinciding with the film thickness at the end of the calcination process. Combining the plasma time during deposition and the annealing temperature post-deposition, control over the crystal size can be obtained and tailored to meet specific application requirements, e.g., for photocatalytic [75] or sensing applications [76–77]. On the zincone-like layer deposited with 1 s plasma exposure time crystallite formation began at 400 °C, as mentioned before, highlighting the role of this temperature range in the possible clogging of porosity. The relative increase in *D* is of 10 nm, contributing to the large increase in refractive index as measured by SE at similar temperatures (Figure 3b).

#### Ellipsometric porosimetry (EP)

**Figure 7:**
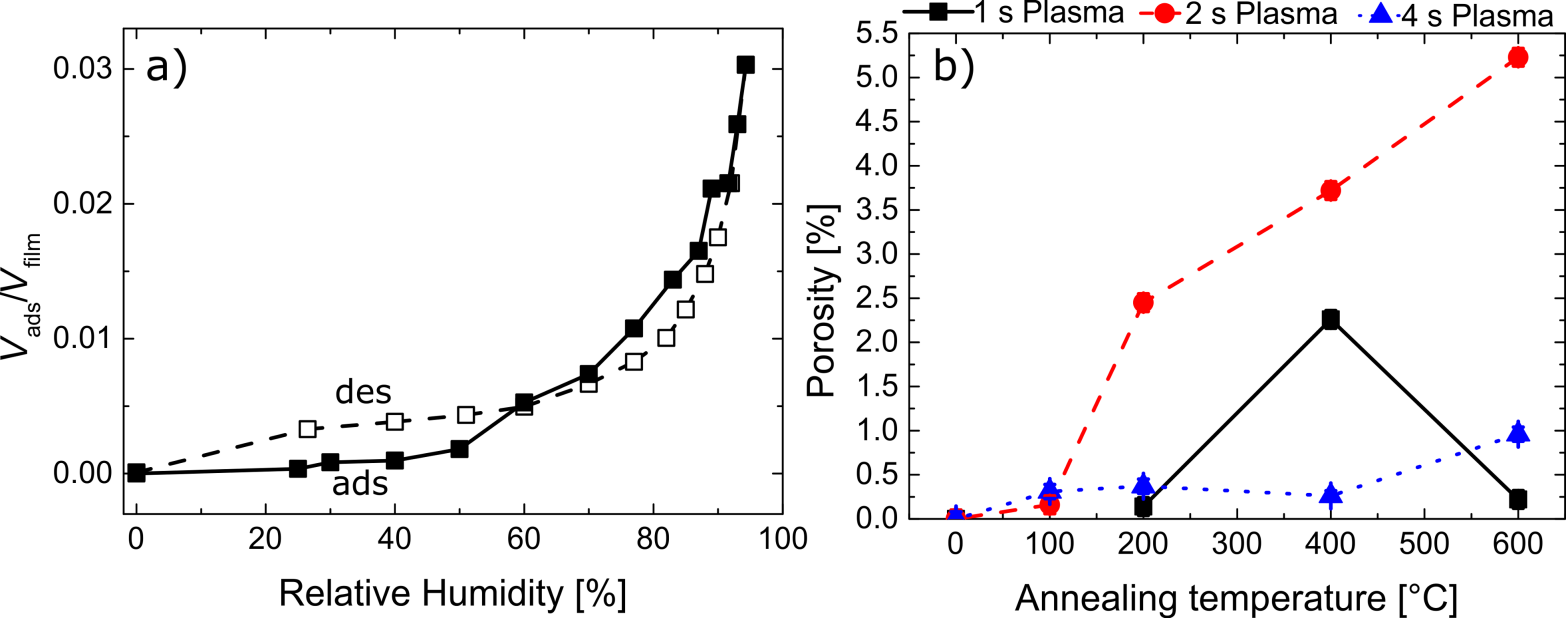
a) Example of an adsorption/desorption isotherm reported as volume of the adsorptive (*V*_ads_/*V*_film_) as a function of the relative humidity for the zincone-like layers; the example is reported for the layer deposited with 1 s plasma exposure time and annealed at 400 °C. b) Porosity values for the zincone-like layers as a function of the annealing temperature.

Finally, in order to verify the open porosity of the layers, EP measurements adopting water as probing molecules were performed (see also Supporting Information File 1). In Figure 7, an example of the adsorption/desorption isotherm and the porosity calculated with Equation 3 as a function of the annealing temperature are reported.

The example reported in Figure 7a shows the two phenomena occurring upon exposure of the annealed zincone-like layers to water vapor. The example is reported for the layer deposited with 1 s plasma exposure time and annealed at 400 °C. In the adsorption branch, two water uptake stages in the formed porosity were identified at 20% and 60% relative humidity. In the literature, these uptakes were attributed to filling of micropores and mesopores, respectively [49–50]. At 90% relative humidity a rapid increase of the water uptake was witnessed. This was attributed to swelling of the layers, accounting for the desorption branch crossing the adsorption branch while decreasing the relative humidity. When water leaves the swollen film, changes in the structure are witnessed with SE, accounting for the lower adsorptive volume in the desorption branch. Swelling of the layers was measured for the layers deposited with 1 s and 2 s plasma exposure time up to 200 °C of annealing temperature. At higher temperatures, as previously stated, burning of the organic functionalities reduces the amount of carbon in the layers and, in turn, the swelling upon water exposure. In the literature, mesoporous materials showed hysteresis in the adsorption and desorption branches [78]. For the zincone-like layers, despite the increase in the refractive index at 60% and 80% relatice humidity, no or limited hysteresis was witnessed. Under these conditions, the pores are generally on the edge between microporosity and mesoporosity, that is, of the order of 1–3 nm in pore diameter. The amount of porosity measured in the layers is reported in Figure 7b. The layers deposited with high plasma exposure times were found to be nonporous over the annealing temperature range explored. Layers deposited at lower plasma exposure times showed instead some residual porosity after calcination. Layers deposited with 1 s plasma exposure time showed a development of porosity between 200 and 400 °C, up to a limit of 2.3%. Increasing the temperature further led to clogging of the created porosity occurring through the collapsing of the ZnO backbone and the increase in crystallite size. The final refractive index value was 1.847 ± 0.003 (Figure 3b), close to the one of pure ZnO deposited under saturation conditions (1.858 ± 0.003 at 633 nm), indicating a low amount closed porosity included in the final ZnO matrix. The layers deposited with intermediate plasma exposure times (2 s) showed the development of controlled porosity as a function of the calcination temperature. Open porosity accessible to water molecules was obtained with values in the range of 2.5–5.5%. The refractive index after calcination (1.725 ± 0.003, Figure 3b) and the low amount of carbon (5%, Table 1) suggest the presence of a high amount of closed porosity, not accessible to water molecules. These results suggest that calcination temperature and organic fraction in the Zn-alkoxide layers have a crucial impact on the amount of porosity that can be achieved. In MLD-derived porous ZnO, despite the higher amount of carbon in the polymer backbone, the regular MLD structure and the lower oxidation state of carbon functionalities could lead to a more controlled removal of the organic fraction and, in turn, void formation. Van de Kerckhove et al. [29] recently demonstrated that the heating rate in the calcination of MLD alucone to form porous alumina has a significant impact on the final open porosity, inferring that the control over the process, together with the control over the film composition, are paramount in the manufacturing of MLD/ALD-derived oxides.

## Conclusion

The formation and development of porosity from Zn-based hybrid thin films is demonstrated. Zinc-alkoxide or “zincone”-like layers were grown with sub-saturated PE-ALD using the sequential exposure of oxygen plasma and DEZ. By adopting different times of exposure to O_2_ plasma, the chemical composition of the resulting hybrid materials was varied, yielding zincone-like thin films with different amount of organic fractions. The calcination of the Zn-alkoxide layers was performed in air, up to 600 °C. In situ SE and XRD together with ex situ GIXD were adopted to follow the degradation of the organic matrix and the formation of porous ZnO thin films. Two temperature windows were identified, namely 200–300 °C and 375–475 °C. In the first, partial removal of the organic fraction and initial formation of voids were witnessed for layers deposited with a plasma exposure time of 2 s. Instead, layers deposited with very short plasma exposure times (1 s) showed the collapse of the temperature-stable ZnO backbone, with consequent clogging of the forming porosity. In the second temperature window, an increase in refractive index and the formation and growth of ZnO crystallites was witnessed, indicating that this temperature range is crucial for the formation of highly porous ZnO layers.

By combining plasma exposure time during deposition and annealing temperature, process and post-treatment windows were identified for the manufacturing of porous ZnO. A controlled open porosity in the range of 2.5–5.5% was measured with EP using water as probing molecule. The pore size was in the range of 0.33–2.00 nm. The adsorption/desorption isotherms suggested the presence of pores with diameters close to mesoporosity (i.e., 2 nm). Moreover, the crystal orientation of the resulting ZnO layers can be varied from powder to (100) preferential orientation in the out-of-plane direction, showing the possibility to control the resulting ZnO crystal orientation. The insight obtained in this study could foster further research on Zn-alkoxide-derived porous ZnO, with important applications, e.g., in photocatalysis and biosensing.

## Supporting Information

File 1 Details on the EP measurements, GIXD data recorded after annealing at 600 °C, saturation curves for the PE-ALD ZnO process optimized at room temperature, example of fitting of the HR XPS peak associated to O 1s.
