# Supplementary material for: On the transformation of “zincone”-like into porous ZnO thin films from sub-saturated plasma enhanced atomic layer deposition
Source: Beilstein J Nanotechnol. 2019 Mar 21;10:746–59. doi: 10.3762/bjnano.10.74 (PMC6444448; doi:10.3762/bjnano.10.74)
Supplement: File 1 — Details on the EP measurements, GIXD data recorded after annealing at 600 °C, saturation curves for the PE-ALD ZnO process optimized at room temperature, example of fitting of the HR XPS peak associated to O 1s. [file Beilstein_J_Nanotechnol-10-746-s001.pdf]

BEILSTEIN JOURNAL OF NANOTECHNOLOGY

Supporting Information

for

On the transformation of “zincone”-like into porous ZnO thin films from sub-saturated plasma enhanced atomic layer deposition

Alberto Perrotta, Julian Pilz, Stefan Pachmajer, Antonella Milella and Anna Maria Coclite

Beilstein J. Nanotechnol. **2019,** *10,* 746–759. doi:10.3762/bjnano.10.74

Details on the EP measurements, GIXD data recorded after annealing at 600 °C, saturation curves for the PE-ALD ZnO process optimized at room temperature, example of fitting of the HR XPS peak associated to O 1s

License and Terms: This is a supporting information file under the terms of the Creative Commons Attribution License (http://creativecommons.org/licenses/by/4.0). Please note that the reuse, redistribution and reproduction in particular requires that the authors and source are credited.
The license is subject to the *Beilstein Journal of Nanotechnology* terms and conditions: (https://www.beilstein-journals.org/bjnano)

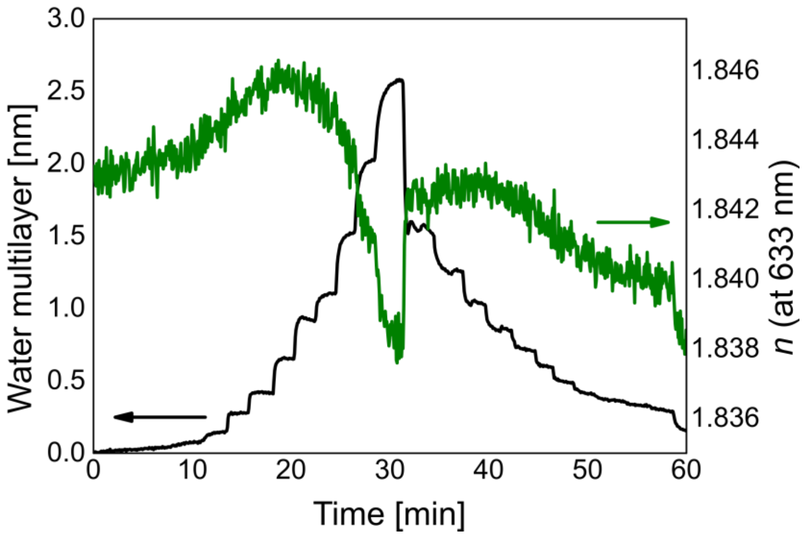

Figure S1: Example of the ellipsometric porosimetry measurement performed on an annealed ZnO layer. The water multilayer thickness and the refractive index are here reported as a function of time.

The relative humidity was changed step by step in the range 0–95%.

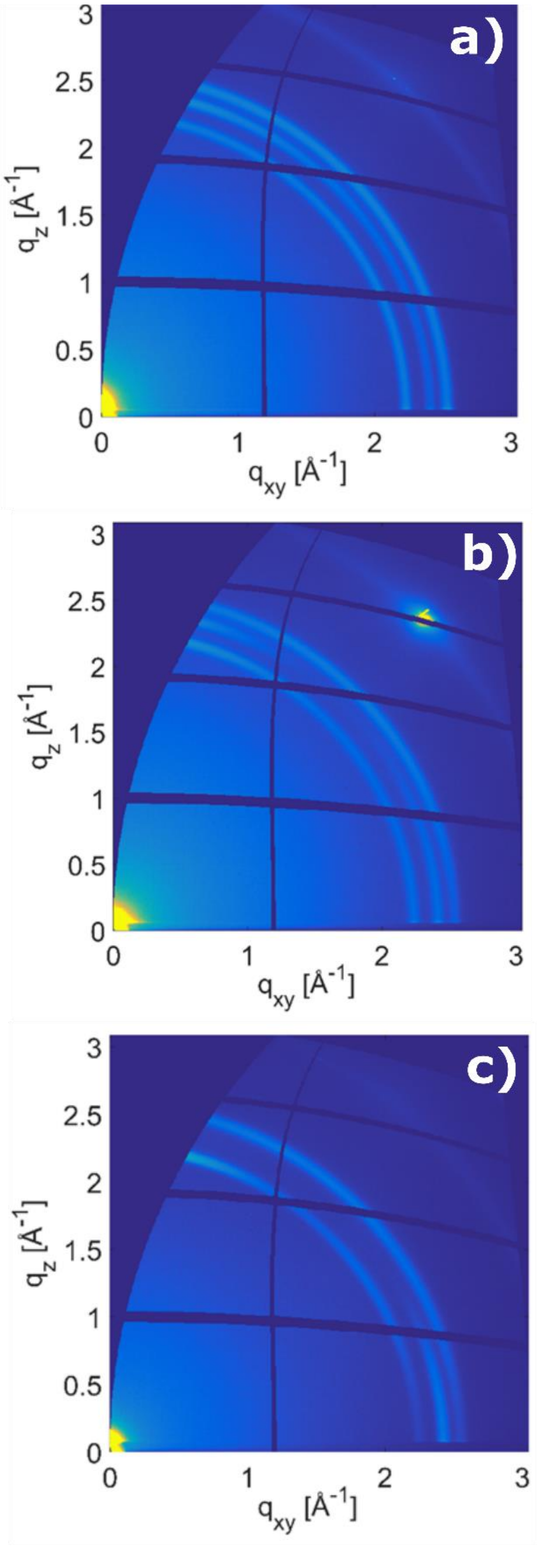

Figure S2: GIXD maps for the zincone-like layers deposited at different plasma times after annealing at 600 °C: a) 1 s b) 2 s and c) 4 s plasma time.

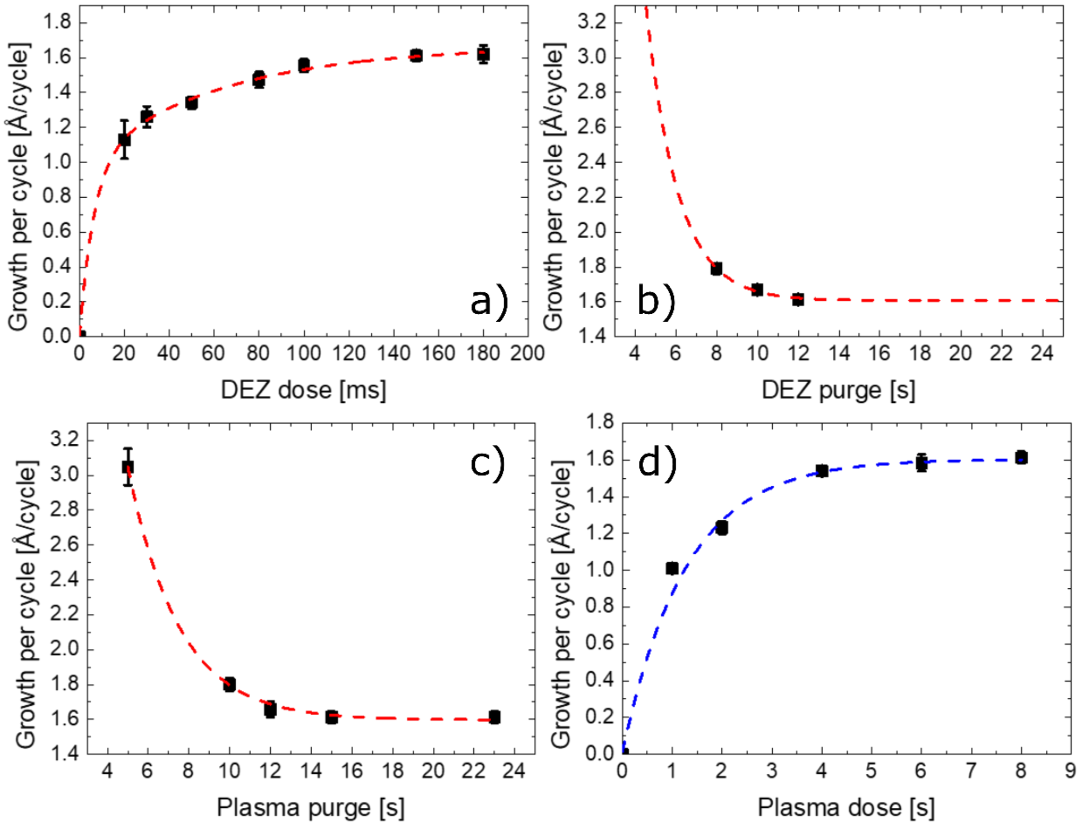

Figure S3: Saturation curves for the PE-ALD ZnO used in this manuscript. a) DEZ dose, b) DEZ purge, c) plasma purge, d) plasma dose, also shown in Figure 1 in the manuscript.

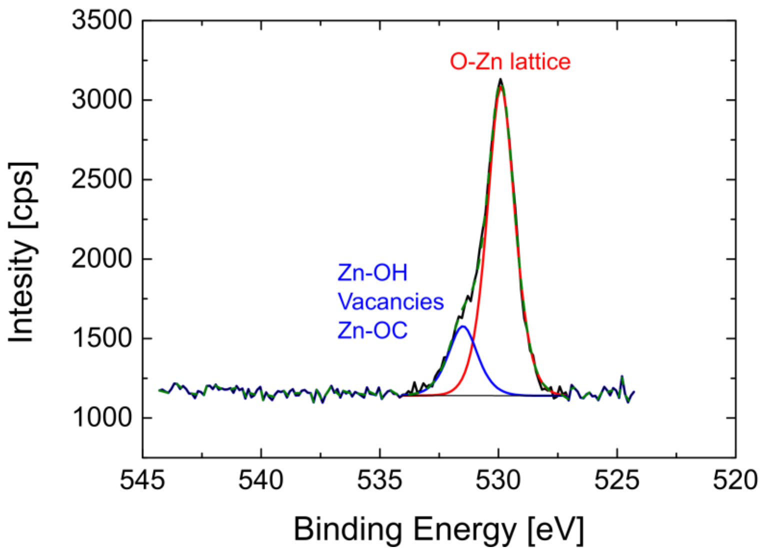

Figure S4: Example of fitting of the HR XPS peak associated to O 1s. the attribution of the peaks is also reported in the plot.
